# Supplementary material for: Visual projection neurons in the Drosophila lobula link feature detection to distinct behavioral programs
Source: eLife. 2016 Dec 28;5:e21022. doi: 10.7554/eLife.21022 (PMC5293491; doi:10.7554/eLife.21022)
Supplement: Supplementary file 1. — (A) Summary of the anatomical properties of the 22 LC neuron types described in detail in this study. Cell numbers and lateral arbor spreads are listed as mean ± SD. Arbors sizes in visual column units were estimated from the measurements of lateral arbor spread. Further details are provided in the Materials and methods. (B) Details of the behavioral experiments in Figures 8–13. This table includes information on split-GAL4 hemidrivers (the AD and DBD halves), the behavioral penetrance for each of the five examined phenotypes, and trial and fly counts, from both the arena and single-fly assays. While the raw data counts within each assay are the same, a small number of trials could not be scored by either manual annotation or automatic tracking; as a result, there are some small differences in the number of quantified data points for the two scoring methods. Use of fly culture media different from standard cornmeal molasses food with supplemental retinal is indicated as follows: ret-: standard cornmeal molasses food without supplemental retinal. Vit-: Vitamin A-deficient food based on the grape juice recipe (see Materials and methods) with supplemental retinal. Vit-, ret-: Vitamin A-deficient food without supplemental retinal. norpA indicates flies that are rendered blind by a null mutation in the norpA gene, norpA[36]. (C) Split-GAL4 driver lines for the LC neuron types described in this study. Split-GAL4 line names in bold indicate drivers used in behavioral experiments. (D) Details of the fly lines used to generate the data for the anatomy Figures and Videos 1 and 2. DOI: http://dx.doi.org/10.7554/eLife.21022.042 [file elife-21022-supp1.docx]

**Supplementary File 1A**

| Cell type | Cell body position and number | Layer pattern and presynaptic sites in the lobula | Size and shape of arbors in the lobula | Axonal projections/target regions Additional anatomical features |
| --- | --- | --- | --- | --- |
| LC4 | Cell bodies in an extended region of the lateral cell body rind (LCBR) near the surface of PVLP and WED (close to or slightly ventral to the LC12 and LC17 glomeruli).  Cell count 66 ± 5 (SS00315, n=4). | Main arbors in layers Lo2 and Lo4. Some processes project into Lo1 and multiple branches pass through Lo3.  ­­­  No evidence for presynaptic sites in the lobula. | Arbor spread in layer Lo2 appears similar along the AP and DV axes (AP: 23 ± 3%, n=13; DV: 22 ± 3%, n=13). Arbors appear slightly narrower in Lo4.  Arbor size in visual column units ~40. Processes of adjacent cells overlap. | Part of a group of ventral glomeruli that includes (from lateral to medial) LC17, LC12, LC18, LPLC2, LC4 and LPLC1. Closest neighbors are LPLC1 and LPLC2.  Axon bundle projects from the medial lobula past the posterior side of the LC17 glomerulus and enters the LC4 glomerulus from lateral (similar to LC22, LPLC1 and LPLC2). |
| LC6 | Cell bodies in dorsal LCBR; approximately from the dorsal edge of the lobula to the dorsal edge of the medulla along DV; slightly posterior to the LC6 glomerulus along AP.  Cell count 77 ± 11 (OL0081B, n=3). (OL0081B expression appears to be incomplete - i.e. some LC6 cells may be unlabeled - suggesting actual cell number is probably higher.) | Main arbors in layers Lo4 and Lo5B, some additional processes in Lo3, Lo5A and Lo6.  Apparent presynaptic sites in both Lo4 and Lo5B (similar to LC16). | Larger arbor spread along the AP than along the DV axis (AP: 29 ± 4%, n=12; DV: 14 ± 2%, n=20; numbers are for layer Lo5B). Arbor spread in Lo4 and Lo5B appears similar.  Arbor size in visual column units ~30. Processes of adjacent cells overlap. | Part of a group of dorsal glomeruli that includes (in approximately lateral to medial order) the target regions of LC15, LC25, LC16, LC26, LC6, LC24 and LC9.  Axons follow the Anterior Optic Tract (AOT) to enter the glomerulus from dorsal and medial. |
| LC9 | Cell bodies in the dorsal LCBR (similar to LC6).  Cell count 119 ± 37 (S02645, n=4).  (Variability appears to be due to incomplete labeling of LC9 cells with this driver line in some brains; if so, actual cell numbers are probably closer to the higher counts of ~ 150.) | Processes in layers Lo2 to Lo5B with the highest arbor density in ~Lo3 and ~Lo5B.  Apparent presynaptic mainly in layers Lo3/Lo4. | Arbor spread (measured in ~ Lo3) is similar along the AP and DV axes (AP: 17 ± 2%, n=9; DV: 14 ± 2%, n=10). Size of Lo5B arbors appears to be similar.  Arbor size in visual column units ~20. Overlap between adjacent cells predicted from cell count and arbor size. | Most medial and anterior of the dorsal PVLP glomeruli (see LC6).  Not closely associated with another glomerulus; adjacent to the more lateral and more posterior LC6.  Axons follow the Anterior Optic Tract (AOT) to enter the glomerulus from dorsal and lateral.  Some retinotopy in the glomerulus (see Figure 4). |
| LC10a | Cell bodies in the dorsal LCBR (similar to LC6).  Cell count 137 ± 7 (OL0019B, n=6). | Main processes in ~Lo3/Lo4 and ~Lo5B. Distal processes extend into Lo2 and there are also some branches in Lo5A and Lo6.  Many apparent presynaptic sites in ~ Lo3. | Arbor spread is similar along the AP and DV axis (AP: 12 ± 3%, n=16; DV: 11 ± 2%, n=14, measured in layer Lo5B) and in Lo5B and Lo3/Lo4.  Arbor size in visual column units ~10. Slight overlap between adjacent cells predicted from cell count and arbor size. | Axons project to the AOTu via the AOT.  Axons fan out near the AOTu to enter the medial zone along most of its DV axis (similar to the LC10B subtype of Otsuna and Ito).  Partial retinotopy in the AOTu (see Figure 4). |
| LC10b | Cell bodies in the dorsal LCBR (similar to LC6).  Cell count ND (No driver line with exclusive expression in this subtype was available.). | Arbors in layers Lo4 to Lo6 with the highest arbor density in Lo6. Many parallel processes project between Lo4 and Lo6.  Many apparent presynaptic sites in Lo6; additional sites in Lo4/Lo5A. | Average arbor spread is the largest of all LC10 subtypes (AP: 30 ± 6%, n=11; DV: 26 ± 5%, n=11, measured in Lo6) but the size of individual LC10b cells appears to be rather variable. Arbor spread in Lo4 and Lo6 is similar.  Arbor size in visual column units ~60. Processes of adjacent cells overlap. | Axons project to the AOTu via the AOT.  Axons enter the medial zone of the AOTu ventrally (similar to the LC10A subtype of Otsuna and Ito).  Partial retinotopy in the AOTu (see Figure 4 legend). |
| LC10c | Cell bodies in the dorsal LCBR (similar to LC6).  Cell count ND (No driver line with exclusive expression in this subtype was available.). | Main arbors in Lo5B; also branches in Lo6 and some processes reaching into Lo5A.  No evidence for presynaptic sites in the lobula. | Arbor spread is similar along the AP and DV axes (AP: 26 ± 4 %, n=7; DV: 24 ± 3%, n=7, measured in Lo5B).  Arbor size in visual column units ~45. Processes of adjacent cells overlap. | Axons project to the AOTu via the AOT.  Axons enter the medial zone of the AOTu ventrally (similar to the LC10A subtype of Otsuna and Ito).  Partial retinotopy in the AOTu (see Figure 4 legend). |
| LC10d | Cell bodies in the dorsal LCBR (similar to LC6).  Cell count 84 ± 4 (S03822, n=4). | Arbors in layers Lo4 to Lo6. The density of processes in Lo5B is lower than that in Lo6 (one difference to LC10a) and in Lo4/Lo5A.  Apparent presynaptic sites in both Lo4 and Lo6; less numerous than those of LC10a and LC10b in Lo3/Lo4 and Lo6, respectively. | Arbor spread similar in Lo4 and Lo6 (AP: 18 ± 3%, n=9; DV: 13 ± 2%, n=8).  Arbor size in visual column units ~15. Processes of adjacent cells overlap.  Note the much smaller arbor size  compared to LC10b. | Axons project to the AOTu via the AOT.  Axons fan out near the AOTu to enter the medial zone along most of its DV axis (similar to the LC10B subtype of Otsuna and Ito).  Partial retinotopy in the AOTu (see Figure 4). |
| LC11 | Cell bodies in the dorsal LCBR (similar to LC6 along DV but more posterior).  Cell count 68 ± 3 (OL0015B, n=3). | Main arborizations in Lo2/Lo3/Lo4 and Lo5B. Lo2 processes do not include the most distal part of this layer.  Processes in Lo5B (mainly near the boundary to Lo5A) are from recurrent branches and appear to have presynaptic sites; additional putative presynaptic sites in ~ Lo4. | Arbor spread in Lo2 (AP: 30 ± 2 %, n=7;DV: 31± 4 %, n=7) is much larger than in Lo5B (AP: 20 ± 4 %, n=7; DV: 15± 3 %, n=7).  Arbor size in visual column units ~70 in Lo2, ~ 25 in Lo5B. Processes of adjacent cells overlap. Cell count and arbor size suggest high coverage (> 5x in Lo2) of lobula columns by LC11 cells. | Elongated PVLP glomerulus located in an intermediate position between the dorsal (see LC6) and ventral (see LC4) groups. Closest neighbor is LC21. Lateral-anterior tip near LC15, close to LC13 at the posterior end.  Axons first project medially in the posterior brain to near the LC13 glomerulus and then turn in an anterior direction to enter the LC11 glomerulus from posterior just anterior to the LC13 glomerulus.  Axons follow the same path as those of LC21. |
| LC12 | Anterior cell body location in the LCBR in an extended region near the LC12/LC17 glomeruli. Together with LC17, the most anterior LC cell bodies.  Cell count 208 ± 14 (OL0008B, n=5). | Main arbors in Lo2 and Lo4; connecting processes and some branches in Lo3. Arbors in Lo2 are absent from the distal part of this layer.  No evidence for presynaptic sites in the lobula. | Arbor spread largest in Lo2 (AP: 14 ± 2%, n=6; DV: 14 ± 1%, n=10); arbors in Lo4 appear narrower along DV axis.  Arbor size in visual column units ~15. Processes of adjacent cells overlap. | Second most lateral of the ventral PVLP glomeruli. Medial and posterior to LC17 and lateral and ventral to LC18. Large glomerulus with a shape (oval in cross-sections) very similar to LC17.  Axons enter glomerulus almost directly after splitting from the dendritic arbors at the base of the lobula. |
| LC13 | Cell bodies in the ventral LCBR (similar to LC16).  Cell count 108 ± 8 (OL0027B, n= 4). | Complex multilayer pattern with arbors in Lo2 to Lo6. Density appears highest in ~ Lo3 and ~Lo5B.  A few apparent presynaptic sites in several lobula layers (mainly ~ Lo3/Lo4). | Arbor spread was measured in ~Lo2/Lo3 (AP: 26 ± 3 %, n=8; DV: 19 ± 4 %, n=8) but may be slightly larger in ~Lo5B.  Arbor size in visual column units ~40 (for Lo2/Lo3). Processes of adjacent cells overlap. | Large, very posterior PVLP glomerulus. Not closely associated with other glomeruli. Anterior tip is close to the posterior parts of the LC21 and also LC11 glomeruli. Together with LC20 the most posterior LC projection.  Axons project dorsally first along the lobula surface and then through the neuropil to reach the glomerulus from ventral and lateral. |
| LC15 | Cell bodies in the ventral LCBR (similar to LC16).  Cell count 70 ± 5 (OL0042B, n=4). | Complex multilayer pattern with the highest arbor densities in ~Lo2/Lo3, ~Lo3/Lo4, ~Lo5A/Lo5B, ~ Lo5B/Lo6; a few branches project into the proximal part of Lo2; branches in Lo6 appear to be mainly in the distal part of this layer.  Apparent presynaptic sites in ~Lo4 and ~ Lo5B. | Arbor spread (AP: 16 ± 3 %, n=15; DV: 19 ± 2 %, n=8, measured in ~Lo2/Lo3) shows some variation between layers.  Arbor size in visual column units ~25 (in ~Lo2/Lo3). Processes of adjacent cells overlap. | Most lateral of the dorsal group (see LC6) of PVLP glomeruli. Close to both this group and LC11 and LC21.  Axons project dorsally from the cell bodies and enter the neuropil at the glomerulus. |
| LC16 | Cell bodies in the ventral LCBR (near the ventral tip of the lobula).  Cell count 96 ± 3 (OL0017B, n=3). | Lobula branches primarily in Lo4 and Lo5B; some processes in Lo5A.  Apparent presynaptic sites in both Lo4 and Lo5B. | Arbor spread was measured in Lo5B (AP: 23 ± 3 %, n=18; DV: 17 ± 3 %, n=20). Some cells have processes in Lo4 that extend beyond the Lo5B spread in a dorsal direction.  Arbor size in visual column units ~30. Processes of adjacent cells overlap. | Part of the dorsal group of PVLP glomeruli (see LC6). Closest neighbors are LC6 and LC26.  Axons project dorsally from the cell bodies and enter the lateral part of glomerulus from ventral and slightly posterior. |
| LC17 | Cell bodies in the anterior LCBR (similar to LC12).  Cell count 181 ± 16 (OL0005B, n=4). | Three main layers: highest arbor density near the Lo2/Lo3 boundary, more diffuse branches near the Lo3/Lo4 boundary and in Lo5B. Lo2 processes do not extend into the most distal part of this layer.  No evidence for presynaptic sites in the lobula. | Arbors in Lo2 (AP: 21 ± 2 %, n=13; DV: 18 ± 2 %, n=11) are wider than those in deeper layers.  Arbor size in visual column units ~30. Processes of adjacent cells overlap. | The most lateral glomerulus of the ventral group (see LC4). Size and shape similar to the more medial and ventral LC12.  Short axon bundle enters glomerulus from posterior. |
| LC18 | Cell bodies distributed along the dorsal posterior surface of the lobula approximately from the base of the LC18 axon bundle to the dorsal tip of the lobula.  Cell count 94 ± 9 (OL0010B, n=5) | The main arbors are in the proximal part of Lo2 near the boundary with Lo3 and in a second layer along the Lo3/Lo4 boundary.  Apparent presynaptic sites in ~Lo3/Lo4. | Distal arbors (AP: 25 ± 5 %, n=7; DV: 17 ± 4 %, n=3, ~Lo2) appear slightly larger than those in ~Lo3/Lo4.  Arbor size in visual column units ~30. Processes of adjacent cells overlap. | Part of the ventral group of PVLP glomeruli (see LC4). Medial to LC12 and lateral to LPLC2, slightly dorsal to both.  Axonal bundle runs from the posterior edge of the lobula to the glomerulus at approximately the same DV position as the glomerulus. |
| LC20 | Cell bodies in the anterior LCBR near the LC15 glomerulus.  Cell count 29 ± 1 (SS00343, n=4). | Main arbors in two layers, Lo5A and Lo6; minor branches in ~ Lo4 and in Lo5B.  Apparent presynaptic sites in both Lo5A and Lo6. | Arbor spread in Lo5A (AP: 23 ± 5 %, n=8; DV: 23 ± 3 %, n=6) and Lo6 appears similar. Processes in ~Lo4 consist of narrow individual branches that are distributed across the lobula but with incomplete coverage.  Arbor size in visual column units ~40. Processes of adjacent cells overlap. | Small, very posterior glomerulus not closely associated with other LC target regions. The glomerulus appears to be somewhat variable in shape; in some brains, it has a bifurcated appearance when viewed along the DV axis.  Axon bundle runs in a posterior medial direction starting just posterior to the LC15 glomerulus. |
| LC21 | Cell bodies distributed along the posterior medial surface of the lobula; approximately from the base of the LC11 axon bundle to the dorsal tip of the lobula along DV.  Cell count 87 ± 2 (SS17690, n=3). | Arbors in four main strata: two closely spaced layers in the distal lobula (~Lo2/Lo3 and ~Lo3/Lo4) and along the boundaries of Lo5B (~Lo5A/Lo5B and ~Lo5B/Lo6). Arbor density highest in the distal layers. Lo2 processes do not extend into the most distal part of this layer.  Apparent presynaptic sites in Lo3/Lo4 and along both boundaries of Lo5B. | Arbor spread in Lo2 (AP: 20 ± 3 %, n=9; DV: 21 ± 4 %, n=8) is similar to deeper layers.  Arbor size in visual column units ~30. Processes of adjacent cells overlap. | Elongated PVLP glomerulus located in an intermediate position between the dorsal (see LC6) and ventral (see LC4) groups.  Closest neighbor is LC11. Lateral-anterior tip near LC15, close to LC13 at the medial posterior end.  Axons follow the same path as those of LC11. |
| LC22 | Cell bodies in the dorsal LCBR (more posterior than LC6, similar to LPLC2).  Cell count 31 ± 3 (OL0001B, n=8). | Main arbors in ~Lo4/Lo5A and Lo6. Connecting processes (several per cell) between these layers pass through (and perhaps have branches in) Lo5B.  Apparent presynaptic sites mainly in ~Lo4 and ~Lo6. | Arbors are elongated along the DV axis (AP: 19 ± 4 %, n=8; DV: 36 ± 12 %, n=10, measured in Lo4). Arbor spread appears similar in Lo4/Lo5A and Lo6.  Arbor size in visual column units ~50. Processes of adjacent cells overlap. | Posterior to LPLC1 but anterior to LC13 and LC20. Not closely associated with other glomeruli except LPLC4 (see below). Nearest other neighbor is LPLC1. More medial than the other glomeruli.  The dorsal side of the LC22 target region is less sharply defined than the boundaries of most other glomeruli. Based on single cell data (see Figure 3- figure supplement 2), there is at least some overlap between the LC22 and LPLC4 target regions.  Axonal path similar to LC4, LPLC1 and LPLC2. |
| LC24 | Cell bodies in the ventral LCBR (similar to LC16).  Cell count 38 ± 6 (SS02638, n=4). | Arbors mainly in Lo5 and Lo6 with some processes into Lo4.  Apparent presynaptic sites sparsely distributed across the lobula arbors. | Arbors are elongated along the DV axis (AP: 18 ± 3 %, n=8; DV: 26 ± 3 %, n=6, measured in Lo5B).  Arbor size in visual column units ~35. Slight overlap between adjacent cells predicted from cell count and arbor size.  General arbor shape variable; main neurite runs in an approximately dorsal direction with many side branches. | Small glomerulus in the dorsal group (see LC6).  Close to LC6/LC16/LC26 at the lateral end but extends to more posterior and medial.  Axons project dorsally from the cell bodies and enter the neuropil near the glomerulus (similar to LC15, LC16, LC25 and LC26). |
| LC25 | Cell bodies in the ventral LCBR (similar to LC16).  Cell count 45 ± 8 (SS02650, n=4). | Arbors primarily in single layer near the Lo5A/Lo5B boundary, some processes extend deeper into Lo5B and into Lo5A.  Apparent presynaptic sites in main layer. | Very large arbor spread compared to most other LC cell types (AP: 46 ± 7 %, n=10; DV: 34 ± 8 %, n=11, measured in Lo5B).  Arbor size in visual column units ~120. Processes of adjacent cells overlap. Cell count and arbor size suggest high coverage (> 5x) of lobula columns by LC25 cells. | Unusual target region with axonal branches near the surface of the LC15 and also the LC6/LC16/LC26 glomeruli. Only cell type in the dorsal group (see LC6) whose target region is not readily recognizable as a distinct region in the pattern of the anti-Brp neuropil marker.  Axons project dorsally from the cell bodies and enter the neuropil near the glomerulus (similar to LC15, LC16, LC24 and LC26). |
| LC26 | Cell bodies in the ventral LCBR (similar to LC16).  Cell count 37 ± 8 (SS02445, n=4). | Arbors mainly in in Lo4 and Lo5B; minor processes in Lo4, Lo5A and Lo6.  No evidence for presynaptic sites in the lobula. | Large arbors in Lo5B (AP: 32 ± 3 %, n=6; DV: 24 ± 5 %, n=4).  Arbor size in visual column units ~60. Overlap between adjacent cells predicted from cell count and arbor size. | Small glomerulus in the dorsal group (see LC6); slightly posterior to its closest neighbors LC6, LC16 and LC25. Lateral and medial tips close to LC15 and LC24, respectively.  Axonal path similar to LC15, LC16, LC24 and LC25. |
| LPLC1 | Cell bodies in the dorsal LCBR (more anterior than LPLC2).  Cell count 62 ± 9 (OL0029B, n=4). | Lobula arbors in Lo2 to Lo4 (with lower density in Lo3) and in Lo5B.  Apparent presynaptic sites in ~ Lo3/Lo4.  Processes also extend through the full depth of the lobula plate but appear to be sparser in Lp1 compared to the other Lp layers. | Lateral arbor spread is similar in Lo2 (AP: 23 ± 2 %, n=6; DV: 15 ± 2 %, n=8) and other lobula layers with LPLC1 processes.  Arbor size in visual column units in the lobula ~25. Lobula arbors of adjacent cells overlap. | Most posterior glomerulus in the ventral group (see LC4); located at the PVLP/PLP boundary. Anterior to LC22.  Closest neighbors are LPLC2 and LC4.  Axonal path similar to LC4, LC22 and LPLC2.  Most cells have three connecting neurites between lobula and lobula plate. |
| LPLC2 | Cell bodies in the dorsal LCBR (more posterior than LC6, more medial and dorsal than LC18).  Cell count 81 ± 4 (OL0048B, n=4). | Processes in the lobula primarily in two layers, Lo4 and Lo5B. Minor branches in Lo5A.  Apparent presynaptic sites mainly in Lo4.  LPLC2 cells also arborize in all four lobula plate layers. | Lobula arbors are more elongated along the DV than along the AP axis (AP: 16 ± 2 %, n=6; DV: 21 ± 3 %, n=5; measured in Lo4). Arbor spread is similar in Lo4 and Lo6.  Arbor size in visual column units in the lobula ~25. Lobula arbors of adjacent cells overlap.  Lobula plate processes show layer specific differences in arbor orientation. | Part of the ventral group of PVLP glomeruli (see LC4). Adjacent to LC18, LC12, LC4 and LPLC1.  Axonal path similar to LC4, LC22 and LPLC1. |

**Supplementary File 1B**

| Driver | Cell type | AD in *attP40* (or  *VK00027*) | DBD  in *attP2* | Arena assay | | | | | | | Single-fly assay | | | | | | |
| --- | --- | --- | --- | --- | --- | --- | --- | --- | --- | --- | --- | --- | --- | --- | --- | --- | --- |
|  |  |  |  | Reaching | Jumping | Reaching and jumping trial count | Forward walking | Backward walking | Turning | Walking and turning trial count | Reaching | Reaching  fly count | Jumping | Forward walking | Backward walking | Turning | Non-reaching  fly count |
| pBDPGAL4U | Control | - | - | 0% | 1% | 144 | 3% | 3% | 3% | 110 | 11% | 66 | 1% | 4% | 0% | 2% | 72 |
| SS00315 | LC4 | R47H03 | R72E01 | 0% | 8% | 165 | 3% | 11% | 5% | 115 | 0% | 51 | 100% | 4% | 4% | 2% | 50 |
| OL0077B | LC6 | R92B02 | R41C07 | 0% | 97% | 108 | 6% | 23% | 16% | 79 | 0% | 39 | 95% | 6% | 14% | 5% | 38 |
| SS02651 | LC9 | VT032961 | VT040569 | 0% | 0% | 145 | 16% | 29% | 4% | 107 | 4% | 48 | 0% | 0% | 31% | 13% | 51 |
| OL0020B (LC10-1) | LC10 | R35D04 | R80E07 | 92% | 0% | 130 | 0% | 32% | 8% | 105 | 100% | 88 | 0% | 0% | 0% | 0% | 92 |
| OL0023B (LC10-2) | LC10 | R72C08 | R50D07 | 0% | 0% | 115 | 16% | 1% | 9% | 80 | 2% | 41 | 0% | 2% | 0% | 2% | 43 |
| OL0015B | LC11 | R22H02 | R20G06 | 1% | 0% | 140 | 24% | 1% | 0% | 103 | 0% | 61 | 2% | 2% | 7% | 3% | 60 |
| OL0008B | LC12 | R35D04 | R55F01 | 0% | 0% | 90 | 14% | 1% | 8% | 86 | 1% | 74 | 1% | 0% | 39% | 3% | 76 |
| OL0027B | LC13 | R14A11 | R50C10 | 0% | 1% | 140 | 5% | 12% | 2% | 102 | 0% | 40 | 15% | 0% | 18% | 3% | 52 |
| OL0042B | LC15 | R26A03 | R24A02 | 0% | 82% | 120 | 15% | 18% | 19% | 84 | 0% | 28 | 28% | 0% | 29% | 7% | 40 |
| OL0046B | LC16 | R26A03 | R54A05 | 0% | 9% | 70 | 0% | 97% | 35% | 65 | 1% | 114 | 18% | 0% | 66% | 19% | 148 |
| OL0005B | LC17 | R21D03 | R65C12 | 0% | 7% | 114 | 62% | 8% | 30% | 71 | 2% | 33 | 21% | 6% | 48% | 42% | 47 |
| OL0010B | LC18 | R92B11 | R82D11 | 0% | 0% | 85 | 9% | 18% | 2% | 57 | 0% | 75 | 10% | 0% | 17% | 3% | 90 |
| SS00343 | LC20 | R17A04  (*VK00027*) | R35B06 | 1% | 1% | 125 | 8% | 7% | 11% | 92 | 4% | 46 | 0% | 0% | 4% | 0% | 48 |
| OL0044B | LC21 | R55C04 | R85F11 | 0% | 1% | 120 | 0% | 20% | 0% | 81 | 0% | 34 | 3% | 0% | 6% | 3% | 37 |
| OL0001B | LC22 | R64G10 | R35B06 | 0% | 1% | 170 | 2% | 40% | 3% | 127 | 21% | 72 | 0% | 0% | 11% | 7% | 75 |
| SS02638 | LC24 | VT038216 | VT026477 | 0% | 1% | 135 | 74% | 0% | 23% | 103 | 15% | 145 | 0% | 9% | 3% | 2% | 153 |
| SS02650 | LC25 | VT009792 | VT002021 | 0% | 2% | 150 | 20% | 4% | 3% | 102 | 0% | 39 | 7% | 0% | 3% | 0% | 43 |
| SS02445 | LC26 | VT007747 | R85H06 | 0% | 3% | 100 | 16% | 2% | 4% | 91 | 3% | 39 | 0% | 0% | 0% | 5% | 40 |
| OL0029B | LPLC1 | R64G09 | R37H04 | 0% | 17% | 133 | 22% | 18% | 30% | 98 | 0% | 72 | 100% | 0% | 0% | 0% | 75 |
| OL0048B | LPLC2 | R19G02 | R75G12 | 0% | 57% | 150 | 16% | 52% | 20% | 95 | 0% | 103 | 88% | 0% | 67% | 8% | 104 |
| OL0046B split half AD | LC16 | R26A03 | - | - | - | - | - | 1% | 3% | 77 | - | - | - | - | - | - | - |
| OL0046B split half DBD | LC16 | - | R54A05 | - | - | - | - | 1% | 5% | 74 | - | - | - | - | - | - | - |
| OL0077B split half AD | LC6 | R92B02 | - | - | - | - | - | - | - | - | - | - | 0% | - | - | - | 60 |
| OL0077B split half DBD | LC6 | - | R41C07 | - | - | - | - | - | - | - | - | - | 0% | - | - | - | 45 |
| OL0020B split half AD | LC10 | R35D04 | - | - | - | - | - | - | - | - | 8% | 39 | - | - | - | - | - |
| OL0020B split half DBD | LC10 | - | R80E07 | - | - | - | - | - | - | - | 21% | 39 | - | - | - | - | - |
| pBDPGAL4U (norpA) | Control | - | - | - | - | - | 3% | 3% | 3% | 66 | 17% | 24 | 28% | - | - | - | 43 |
| OL0046B (norpA) | LC16 | R26A03 | R54A05 | - | - | - | - | 87% | 42% | 78 | - | - | - | - | - | - | - |
| OL0077B (norpA) | LC6 | R92B02 | R41C07 | - | - | - | - | - | - | - | - | - | 85% | - | - | - | 33 |
| OL0020B (norpA) | LC10 | R35D04 | R80E07 | - | - | - | - | - | - | - | 100% | 27 | - | - | - | - | - |
| OL0046B (ret-) | LC16 | R26A03 | R54A05 | - | - | - | - | 18% | 2% | 87 | - | - | - | - | - | - | - |
| OL0077B (ret-) | LC6 | R92B02 | R41C07 | - | - | - | - | - | - | - | - | - | 0% | - | - | - | 85 |
| pBDPGAL4U (Vit-) | Control | - | - | - | - | - | - | - | - | - | 22% | 37 | - | - | - | - | - |
| pBDPGAL4U (Vit-, ret-) | Control | - | - | - | - | - | - | - | - | - | 15% | 33 | - | - | - | - | - |
| OL0020B (Vit-) | LC10 | R35D04 | R80E07 | - | - | - | - | - | - | - | 98% | 64 | - | - | - | - | - |
| OL0020B (Vit-, ret-) | LC10 | R35D04 | R80E07 | - | - | - | - | - | - | - | 40% | 35 | - | - | - | - | - |
| SS02664 (Vit-) | LC10 | R35D04 | VT043656 | - | - | - | - | - | - | - | 100% | 14 | - | - | - | - | - |
| SS02664 (Vit-, ret-) | LC10 | R35D04 | VT043656 | - | - | - | - | - | - | - | 9% | 22 | - | - | - | - | - |
| OL0046B | LC16 | R26A03 | R54A05 | - | - | - | - | 94% | 36% | 89 | - | - | - | - | - | - | - |
| OL0017B | LC16 | R82D11 | R54A05 | - | - | - | - | 95% | 29% | 94 | - | - | - | - | - | - | - |
| OL0092C | LC16 | R92B02 (*VK00027*) | R38E08 | - | - | - | - | 89% | 35% | 65 | - | - | - | - | - | - | - |
| OL0202B | LC6 | R42E06 | VT019053 | - | - | - | - | - | - | - | - | - | 100% | - | - | - | 44 |
| OL0203B | LC6 | R42E06 | VT022290 | - | - | - | - | - | - | - | - | - | 91% | - | - | - | 47 |
| OL0218B | LC6 | R47E07 | VT049344 | - | - | - | - | - | - | - | - | - | 100% | - | - | - | 55 |
| OL0070B | LC6 | R91B01 | R22A07 | - | - | - | - | - | - | - | - | - | 96% | - | - | - | 49 |
| OL0071B | LC6 | R91B01 | R41C07 | - | - | - | - | - | - | - | - | - | 90% | - | - | - | 51 |
| OL0081B | LC6 | R47E07 | R41C07 | - | - | - | - | - | - | - | - | - | 80% | - | - | - | 41 |
| OL0087B | LC6 | R82D11 | R41C07 | - | - | - | - | - | - | - | - | - | 93% | - | - | - | 29 |
| OL0019B | LC10 | R35D04 | R22D06 | - | - | - | - | - | - | - | 67% | 92 | - | - | - | - | - |
| OL0021B | LC10 | R35D04 | R82D11 | - | - | - | - | - | - | - | 86% | 43 | - | - | - | - | - |
| OL0022B | LC10 | R35D04 | R91B01 | - | - | - | - | - | - | - | 92% | 24 | - | - | - | - | - |
| SS00937 | LC10 | R22A07 | R35D04 | - | - | - | - | - | - | - | 22% | 41 | - | - | - | - | - |
| SS00938 | LC10 | R35D04 | R71E06 | - | - | - | - | - | - | - | 94% | 51 | - | - | - | - | - |
| SS00940 | LC10 | R35D04 | R22A07 | - | - | - | - | - | - | - | 9% | 44 | - | - | - | - | - |
| SS00941 | LC10 | R47E07 (*VK00027*) | R35D04 | - | - | - | - | - | - | - | 2% | 47 | - | - | - | - | - |
| SS00942 | LC10 | R47E07 | R35D04 | - | - | - | - | - | - | - | 0% | 27 | - | - | - | - | - |
| SS02663 | LC10 | R35D04 | VT040747 | - | - | - | - | - | - | - | 54% | 95 | - | - | - | - | - |
| SS02664 | LC10 | R35D04 | VT043656 | - | - | - | - | - | - | - | 100% | 40 | - | - | - | - | - |
| SS02665 | LC10 | R35D04 | VT058944 | - | - | - | - | - | - | - | 2% | 55 | - | - | - | - | - |
| SS02666 | LC10 | R35D04 | VT045997 | - | - | - | - | - | - | - | 72% | 39 | - | - | - | - | - |
| SS02669 | LC10 | R72C08 | VT040747 | - | - | - | - | - | - | - | 24% | 84 | - | - | - | - | - |
| SS02670 | LC10 | R72C08 | VT058944 | - | - | - | - | - | - | - | 0% | 48 | - | - | - | - | - |
| SS02681 | LC10 | VT040747 | R72C08 | - | - | - | - | - | - | - | 29% | 58 | - | - | - | - | - |
| SS03822 | LC10 | VT043920 | R91B01 | - | - | - | - | - | - | - | 80% | 30 | - | - | - | - | - |

**Supplementary File 1C**

| Cell type | Driver line | AD in *attP40* (or *VK00027*) | DBD in *attP2* |
| --- | --- | --- | --- |
| LC4 | **SS00315** | R47H03 | R72E01 |
| LC6 | **OL0070B** | R91B01 | R22A07 |
|  | **OL0071B** | R91B01 | R41C07 |
|  | **OL0077B** | R92B02 | R41C07 |
|  | **OL0081B** | R47E07 | R41C07 |
|  | **OL0087B** | R82D11 | R41C07 |
|  | **OL0202B** | R42E06 | VT019053 |
|  | **OL0203B** | R42E06 | VT022290 |
|  | **OL0218B** | R47E07 | VT049344 |
| LC9 | SS02645 | VT027704 | VT040569 |
|  | **SS02651** | VT032961 | VT040569 |
|  | SS02652 | VT032961 | VT027704 |
|  | SS02662 | R28G03 | VT027704 |
| LC10-group  (for subtype expression see Figure 10 – figure supplement 2) | **OL0019B** | R35D04 | R22D06 |
|  | **OL0020B** | R35D04 | R80E07 |
|  | **OL0021B** | R35D04 | R82D11 |
|  | **OL0022B** | R35D04 | R91B01 |
|  | **OL0023B** | R72C08 | R50D07 |
|  | **SS00937** | R22A07 | R35D04 |
|  | **SS00938** | R35D04 | R71E06 |
|  | **SS00940** | R35D04 | R22A07 |
|  | SS00941 | R47E07 (*VK00027*) | R35D04 |
|  | SS00942 | R47E07 | R35D04 |
|  | **SS02663** | R35D04 | VT040747 |
|  | **SS02664** | R35D04 | VT043656 |
|  | **SS02665** | R35D04 | VT058944 |
|  | **SS02666** | R35D04 | VT045997 |
|  | **SS02669** | R72C08 | VT040747 |
|  | **SS02681** | VT040747 | R72C08 |
|  | **SS03822** | VT043920 | R91B01 |
| LC11 | **OL0015B** | R22H02 | R20G06 |
| LC12 | OL0007B | R35D04 | R65B05 |
|  | **OL0008B** | R35D04 | R55F01 |
| LC13 | **OL0027B** | R14A11 | R50C10 |
| LC15 | OL0003B | R24A02 | R26A03 |
|  | OL0004B | R24A02 | R91B01 |
|  | **OL0042B** | R26A03 | R24A02 |
| LC16 | **OL0017B** | R82D11 | R54A05 |
|  | **OL0046B** | R26A03 | R54A05 |
|  | **OL0092C** | R92B02 (*VK00027*) | R38E08 |
| LC17 | **OL0005B** | R21D03 | R65C12 |
| LC18 | **OL0010B** | R92B11 | R82D11 |
|  | OL0011B | R82D11 | R92B11 |
| LC20 | **SS00343** | R17A04 (*VK00027*) | R35B06 |
|  | OL0009B | R71F06 | R35B06 |
| LC21 | **OL0044B** | R55C04 | R85F11 |
|  | OL0045B | R55C04 | R41C05 |
|  | SS17690 | R50C03 | R85F11 |
| LC22 | **OL0001B** | R64G10 | R35B06 |
| LC24 | **SS02638** | VT038216 | VT026477 |
| LC25 | **SS02650** | VT009792 | VT002021 |
| LC26 | **SS02445** | VT007747 | R85H06 |
| LPLC1 | **OL0029B** | R64G09 | R37H04 |
| LPLC2 | **OL0048B** | R19G02 | R75G12 |

**Supplementary File 1D**

| **Figure** | **Panel(s)** | **Driver line(s)** | **Reporter transgene(s)** |
| --- | --- | --- | --- |
| 1 | D-I | OL0005B (D,G,H), OL0046B (E,F,I) | pJFRC51-3XUAS-IVS-syt::smHA in *su(Hw)attP1*,pJFRC225-5XUAS-IVS-myr::smFLAG in *VK00005* |
| 1 | J,K | OL0046B | MCFO-1 |
| 1-s1 |  | OL0037B (R87D07-AD; R59B03-DBD) (A), OL0038B (R44A02-AD; R50B11-DBD) (B), OL0012B (R67E03-AD; R94D12-DBD) (C), OL0036B (R87D07-AD; R50B11-DBD) (D), R82F01 (E) | MCFO-1 (B,C,D), MCFO-7 (A,E) |
| 2 |  | As indicated in Figure | 20XUAS-CsChrimson-mVenus in *attP18* |
| 2-s1 |  | As indicated in Figure | 20XUAS-CsChrimson-mVenus in *attP18* |
| 3 | A,B | SS00315 (LC4), OL0070B (LC6), SS02645 (LC9), OL0015B (LC11), OL0008B (LC12), OL0042B (LC15), OL0017B (LC16), OL0005B (LC17), OL0010B (LC18), SS17690 (LC21), OL0029B (LPLC1), OL0048B (LPLC2) | pJFRC51-3XUAS-IVS-syt::smHA in *su(Hw)attP1*,pJFRC225-5XUAS-IVS-myr::smFLAG in *VK00005* |
| 3 | D-F | OL0027B (LC13) (D,F), SS00343 (LC20) (D,F), OL0001B (LC22) (D,F), SS02638 (LC24) (D,E), SS02650 (LC25) (D,E), SS02445 (LC26) (D,E) | pJFRC51-3XUAS-IVS-syt::smHA in *su(Hw)attP1*,pJFRC225-5XUAS-IVS-myr::smFLAG in *VK00005* |
| 3 | G,H | OL0019B (Line1), OL0023B (Line2) | pJFRC51-3XUAS-IVS-syt::smHA in *su(Hw)attP1*,pJFRC225-5XUAS-IVS-myr::smFLAG in *VK00005* |
| 3 | C,I | NA (image shows anti-Brp pattern of standard brain) | NA |
| 3-s1 |  | OL0008B (LC12), OL0010B (LC18), OL0048B (LPLC2), SS00315 (LC4), OL0015B (LC11), SS02650 (LC25), OL0017B (LC16), OL0070B (LC6) | pJFRC51-3XUAS-IVS-syt::smHA in *su(Hw)attP1*,pJFRC225-5XUAS-IVS-myr::smFLAG in *VK00005* |
| 3-s2 |  | R11C10-GAL4 | MCFO-7 |
| 4 | A-C,H-M | OL0017B (A-C), OL0019B (H-J), SS03822 (K-M), | MCFO-1 |
| 4 | D-G | 29C07-KDGeneswitch-4 in attP40; R57C10-GAL4 in attP2, tubP-KDRT>GAL80-6-KDRT> in VK00027 (drives stochastic GAL4 expression in optic lobe neurons; see ([Nern et al., 2015](#_ENREF_1)) for details) | MCFO-1 |
| 4-s1 | A-R | OL0077B (A-C), OL0015B (D-F), OL0027B (G-I), OL0007B (J-L), OL00011B (M-O), OL0048B (P-R) | MCFO-1 |
| 5 | A | NA (image shows anti-Brp pattern of standard brain) | NA |
| 5 | B-D | OL0070B (LC6), OL0008B (LC12), SS00315 (LC4), SS02645 (LC9), OL0015B (LC11), OL0027B (LC13), OL0042B (LC15), OL0046B (LC16), OL0005B (LC17), OL0010B (LC18), SS00343 (LC20), OL0044B (LC21), OL0001B (LC22), SS02638 (LC24), SS02650 (LC25), SS02445 (LC26), OL0029B (LPLC1), OL0048B (LPLC2) | pJFRC51-3XUAS-IVS-syt::smHA in *su(Hw)attP1*,pJFRC225-5XUAS-IVS-myr::smFLAG in *VK00005* |
| 5 | E,F | SS00315 (LC4), OL0003B (LC15), OL0022B (LC10a, LC10d), SS02664 (LC10b), SS00940 (LC10c) | MCFO-1 (LC4, LC15, LC10a, LC10c, LC10d), MCFO-7 (LC10b) |
| 5-s1 |  | 29C07-KDGeneswitch-4 in attP40; R57C10-GAL4 in attP2, tubP-KDRT>GAL80-6-KDRT> in VK00027 (drives stochastic GAL4 expression in optic lobe neurons; see ([Nern et al., 2015](#_ENREF_1)) for details) | MCFO-1 |
| 5-s2 |  | OL0019B (A), OL0015B (B), SS03822 (C), OL0010B (D), SS00941 (E), SS00343 (F), OL0029B (G), OL0048B (H) | pJFRC51-3XUAS-IVS-syt::smHA in *su(Hw)attP1*,pJFRC225-5XUAS-IVS-myr::smFLAG in *VK00005* |
| 6 | A-E | OL0015B (A), OL0019B (B), OL0001B (C), SS00313 (R38C11-AD, R59C10-DBD) (D), VT011128 (E) | MCFO-1 (A,B), MCFO-7 (C-E) |
| 6-s1 |  | SS00315 (LC4), OL0070B (LC6), SS02662 (LC9), SS00937 (LC10b), SS02669 (LC10c), SS03822 (LC10d), OL0008B (LC12), OL0027B (LC13), OL0004B (LC15), OL0046B (LC16), OL0008B (LC17), OL0011B (LC18), SS00343 (LC20), OL0045B (LC21), SS02638 (LC24), SS02650 (LC25), SS02445 (LC26), OL0029B (LPLC1), OL0048B (LPLC2) | MCFO-1 (LC4, LC6, LC10b, LC12, LC13, LC15, LC16, LC17, LC21, LC24, LC25, LC26, LPLC1, LPLC2), MCFO-7 (LC9, LC10c, LC18, LC20) |
| 7 |  | S00315 (LC4), OL0070B (LC6), SS02662 (LC9), OL0019B (LC10a), OL0023B (LC10b), SS00942 (LC10c), SS03822 (LC10d), OL0015B (LC11), OL0008B (LC12), OL0027B (LC13), OL0042B (LC15), OL0046B (LC16), OL0005B (LC17), OL0010B (LC18), SS00343 (LC20), OL0045B (LC21), OL0001B (LC22), SS02638 (LC24), SS02650 (LC25), SLC26), OL0029B (LPLC1), OL0048B (LPLC2) | MCFO-1 (LC4, LC6, LC10a, LC10b, LC10c,  LC10d, LC11, LC12, LC13, LC15, LC17, LC18, LC21, LC24, LC25, LC26, LPLC1, LPLC2), MCFO-7 (LC9, LC16, LC20, LC22) |
| 7-s1 |  | SS00315 (LC4), OL0070B (LC6), SS02652 (LC9), OL0019B (LC10a), SS00937 (LC10b), SS00940 (LC10c), SS03822 (LC10d), OL0015B (LC11), OL0008B (LC12), OL0027B (LC13), OL0003B (LC15), OL0017B (LC16), OL0005B (LC17), OL0010B (LC18), SS00343 (LC20), OL0045B (LC21), OL0001B (LC22), SS02638 (LC24), SS02650 (LC25), SS02445 (LC26), OL0029B (LPLC1), OL0048B (LPLC2) | MCFO-1 (LC4, LC6, LC9, LC10a, LC10b, LC10c, LC10d, LC11, LC12, LC13, LC15, LC16, LC17, LC18, LC21, LC24, LC25, LC26, LPLC1, LPLC2), MCFO-7 (LC20, LC22) |
| 7-s2 |  | OL0029B (A), OL0048B (B), VT058688 (C) | MCFO-1 |
| 9-s1 |  | As indicated in Figure | 20XUAS-CsChrimson-mVenus in *attP18* |
| 10 | B,C (top panel) | As indicated in Figure | pJFRC51-3XUAS-IVS-syt::smHA in *su(Hw)attP1*,pJFRC225-5XUAS-IVS-myr::smFLAG in *VK00005* |
| 10 | C (bottom panels) | SS02664 | MCFO-7 |
| 10-s1 |  | As indicated in Figure | 20XUAS-CsChrimson-mVenus in *attP2* |
| 10-s2 | A,B | As indicated in Figure | MCFO-1, MCFO-7 |
| 11 | A | OL0202B (LC6), OL0046B (LC16) | 20XUAS-CsChrimson-mVenus in *attP18* |
| 11 | B | OL0070B (LC6), OL0046B (LC16) | pJFRC51-3XUAS-IVS-syt::smHA in *su(Hw)attP1*,pJFRC225-5XUAS-IVS-myr::smFLAG in *VK00005* |
| 12 | B | OL0070B (LC6), OL0017B (LC16), OL0015B (LC11) | pJFRC51-3XUAS-IVS-syt::smHA in *su(Hw)attP1*,pJFRC225-5XUAS-IVS-myr::smFLAG in *VK00005* |
| 13 | B | OL0046B | pJFRC300-20XUAS-FRT>-dSTOP-FRT>-CsChrimson-mVenus in *attP18* |
| Movie 1 |  | Same as Figures 3B,D combined | Same as Figure 3 |
| Movie 2 |  | Same as Figures 3B,D combined | Same as Figure 3 |
